# Pseudoachalasia Due to Malignant Pleural Mesothelioma Involving the Esophagus

**DOI:** 10.7759/cureus.84161

**Published:** 2025-05-15

**Authors:** Manami Honda, Masaya Iwamuro, Kazuya Miyamoto, Takehiro Tanaka, Motoyuki Otsuka

**Affiliations:** 1 Department of Gastroenterology and Hepatology, Okayama University Graduate School of Medicine, Dentistry, and Pharmaceutical Sciences, Okayama, JPN; 2 Department of Pathology, Okayama University Hospital, Okayama, JPN

**Keywords:** endoscopic ultrasound-guided fine-needle aspiration, esophageal diseases, esophagogastroduodenoscopy (egd), malignant mesothelioma, pseudoachalasia

## Abstract

We report a rare case of pseudoachalasia secondary to malignant pleural mesothelioma involving the esophagus. A 66-year-old man presented with progressive dysphagia, weight loss, and postprandial hiccups. Endoscopic examination showed esophageal dilation with luminal narrowing at the esophagogastric junction, but no mucosal abnormalities. Computed tomography revealed an irregular-shaped mass extending from the peri-esophagogastric junction to the retroperitoneum, accompanied by pleural effusion, right-sided hydronephrosis, and multiple hepatic lesions. Endoscopic ultrasound-guided fine-needle aspiration from the mass lesion through the esophageal lumen revealed epithelioid malignant mesothelioma. This case highlights the importance of considering malignant mesothelioma in the differential diagnosis of pseudoachalasia, particularly when imaging reveals extrinsic esophageal compression without mucosal lesions.

## Introduction

Achalasia is a rare esophageal motility disorder characterized by impaired relaxation of the lower esophageal sphincter and absent peristalsis, leading to dysphagia and regurgitation [1,2]. CT analysis may reveal esophageal dilatation with an air-fluid level and a tapering “bird-beak” appearance at the esophagogastric junction. Chronic cases can show marked esophageal dilation, wall thickening, or food retention.

Pseudoachalasia, also known as secondary achalasia, is a clinical condition that mimics idiopathic achalasia, characterized by impaired relaxation of the lower esophageal sphincter and esophageal dilatation, typically resulting in progressive dysphagia and weight loss [3-5]. While the majority of pseudoachalasia cases are secondary to malignancies involving the esophagogastric junction, such as gastric adenocarcinoma or esophageal carcinoma, other rare etiologies must be considered [6,7]. Malignant pleural mesothelioma, a neoplasm strongly associated with asbestos exposure, rarely manifests with esophageal involvement leading to pseudoachalasia. Given its aggressive nature and poor prognosis, early and accurate diagnosis is essential [8].

Herein, we report a rare case of pseudoachalasia secondary to malignant pleural mesothelioma, highlighting the diagnostic challenges and the clinical importance of differentiating this condition from idiopathic achalasia.

## Case presentation

A 66-year-old Japanese man presented to his family clinic with a two-month history of unintentional weight loss (8 kg), followed by the onset of dysphagia one month prior to presentation. Two weeks before the presentation, he also developed lower back pain and postprandial hiccups. His medical history included hypertension, gastric ulcer, and cholecystectomy due to cholecystitis. He had no known history of chest diseases. The patient had a smoking history of 100 cigarettes per day for 30 years (150 pack-years) and consumed 350 mL of beer daily for 45 years. His height was 170.8 cm and weight was 45.6 kg. Physical examination revealed stable vital signs, including a blood pressure of 110/67 mmHg, a pulse rate of 74 bpm, and a body temperature of 35.9°C. The neurological examination showed no focal deficits or abnormalities. Examination of the chest and abdomen showed no palpable abnormalities. Lymphadenopathy was not detected.

Laboratory findings revealed an increased uric acid of 8.2 mg/dL (reference range: 3.6-7.0 mg/dL), an amylase level of 148 U/L (40-130 U/L), a hemoglobin A1c level of 6.2% (4.9-6.0%), and soluble interleukin-2 receptor levels of 545 U/mL (122-496 U/mL). The complete blood count and other biochemical parameters, including lactate dehydrogenase, were within normal ranges.

**Figure 1:**
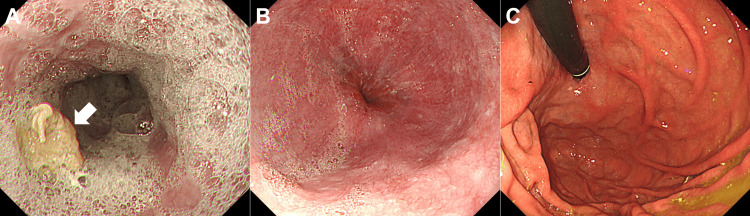
Esophagogastroduodenoscopy findings. Marked esophageal dilatation with retained food residues was observed (A, arrow). Luminal narrowing at the esophagogastric junction was noted without mucosal abnormalities (B). Although the endoscope was able to pass through the stricture, significant resistance was encountered. Retroflexed endoscopic observation within the stomach revealed no detectable tumor in the cardia (C).

**Figure 2:**
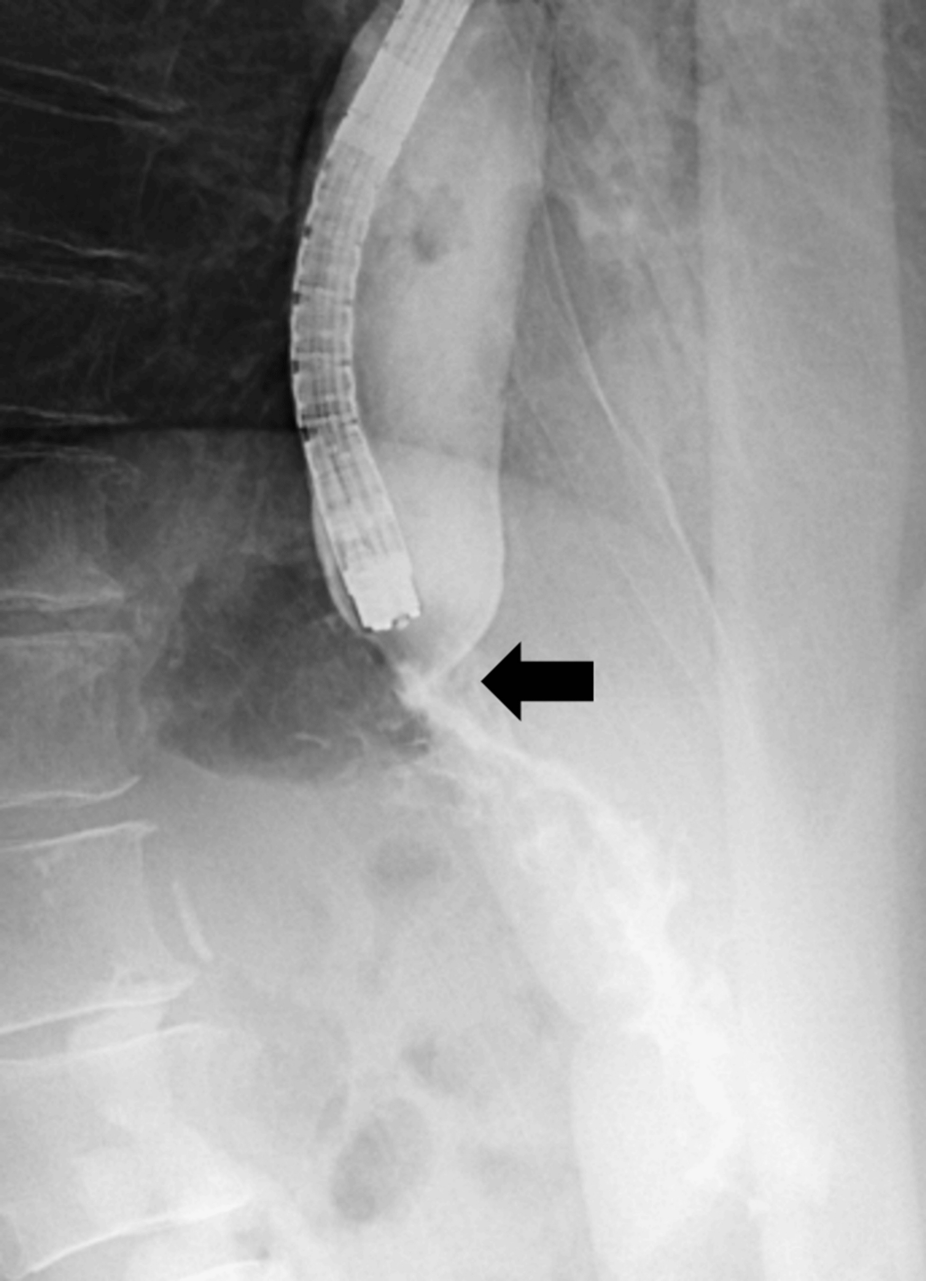
Contrast-enhanced image during endoscopic examination. The arrow indicates a constriction at the esophagogastric junction consistent with the site of luminal narrowing.

**Figure 3:**
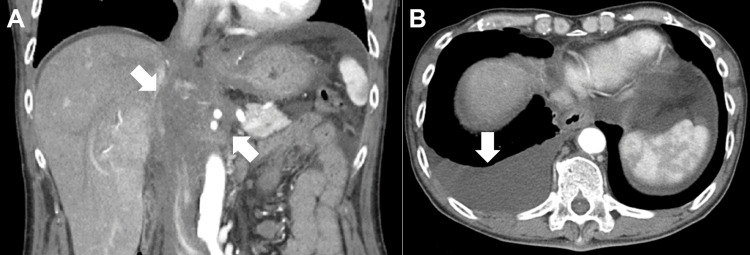
Computed tomography findings. An irregular mass (arrows) extending from the peri-esophagogastric junction to the retroperitoneum was identified (A). Right-sided pleural effusion was observed (B, arrow).

Esophagogastroduodenoscopy revealed significant esophageal dilatation with retained food residues (Fig. 1A). There was notable luminal narrowing at the esophagogastric junction, though no mucosal abnormalities were evident (Fig. 1B, 1C). Although the endoscope was able to pass through, significant resistance was encountered. Scars from previous gastric ulcers were observed on the lesser curvature of the middle gastric body, but no overt neoplastic lesions were identified. Biopsies taken from the esophagogastric junction revealed no evidence of neoplastic cells. Contrast studies performed by injecting a water-soluble iodine-based contrast agent (diatrizoate meglumine and diatrizoate sodium) through the endoscope confirmed the presence of esophageal constriction (Fig. 2, arrow). Computed tomography (CT) revealed an irregular-shaped mass extending from the peri-esophagogastric junction to the retroperitoneum (Fig. 3A, arrows), accompanied by pleural effusion (Fig. 3B), right-sided hydronephrosis, and multiple hepatic lesions.

**Figure 4:**
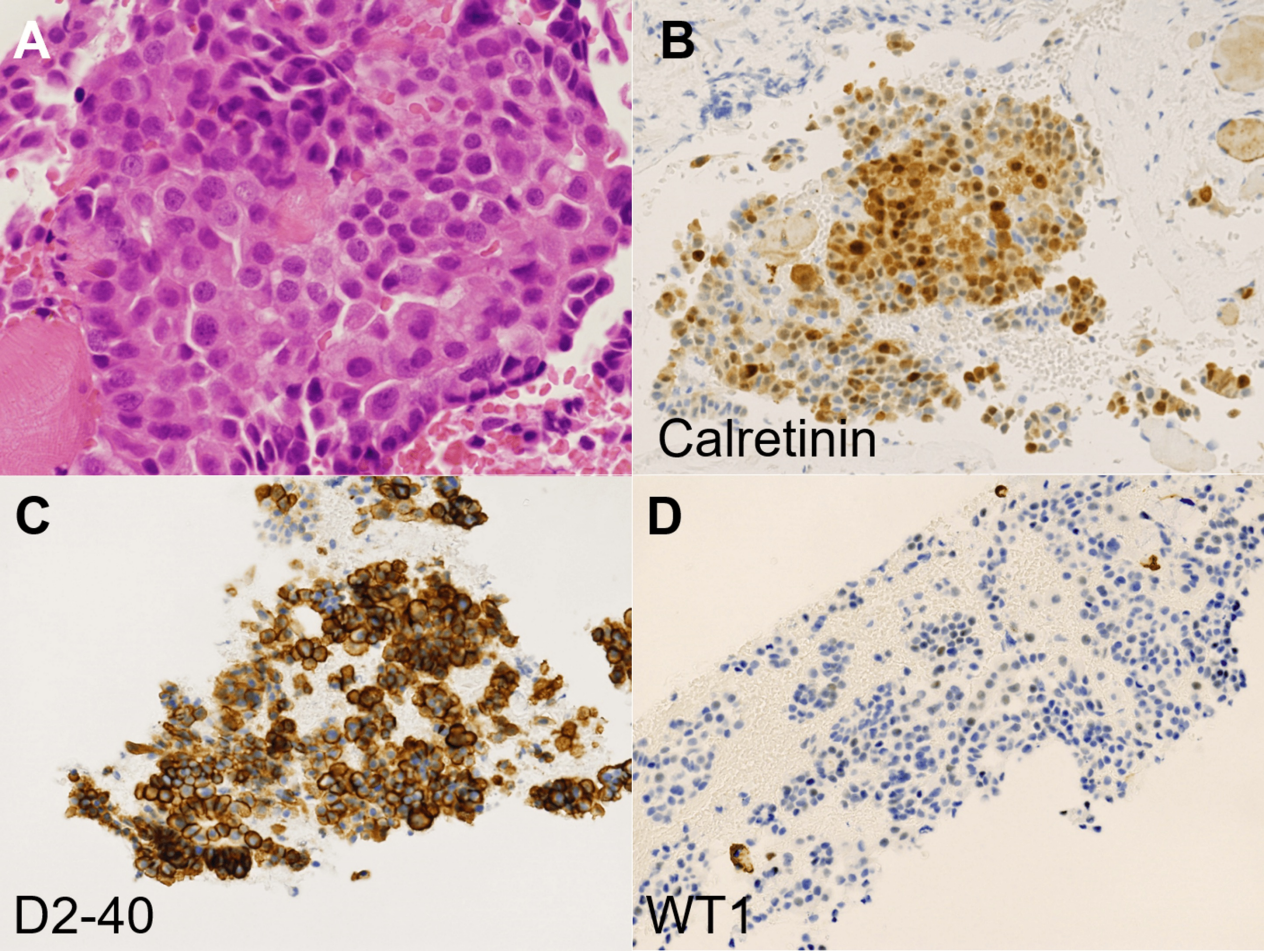
Histopathological and immunohistochemical analysis of the mass obtained by endoscopic ultrasound-guided fine-needle aspiration. Small, round neoplastic cells proliferating in a sheet-like pattern (A, Hematoxylin and Eosin staining, ×40). The tumor cells were positive for calretinin (B, ×20). Positive staining for podoplanin (C, ×20).  Sparse positivity for Wilms' tumor 1 (WT1) (D, ×20).

To establish the diagnosis, endoscopic ultrasound-guided fine-needle aspiration (EUS-FNA) was performed using a linear echoendoscope (GF-UCT260, Olympus, Tokyo, Japan) on the mass extending from the peri-esophagogastric junction to the retroperitoneum. Pathological analysis revealed small, round neoplastic cells (Fig. 4A) positive for calretinin (Fig. 4B) and podoplanin (Fig. 4C), with sparse positivity for Wilms' tumor 1 (Fig. 4D), and negative results for synaptophysin, c-kit, and α-smooth muscle actin. These immunohistochemical markers confirmed the diagnosis of epithelioid malignant mesothelioma. Further supporting evidence was obtained from pleural effusion cytology, which revealed similar neoplastic cells, thereby confirming the diagnosis of malignant pleural mesothelioma with esophageal involvement. A reassessment of the patient's occupational history revealed prior work in piping construction at the age of 30, with a potential asbestos exposure.

Following the diagnosis, the patient was started on a chemotherapy regimen consisting of pemetrexed and cisplatin, the standard first-line treatment for malignant pleural mesothelioma, which was administered for five cycles. Due to obstruction at the esophagogastric junction, a gastrostomy was performed to provide nutritional support. After disease progression, the patient became refractory to the initial chemotherapy and subsequently received 13 cycles of nivolumab. This was followed by three cycles of gemcitabine, five additional cycles of nivolumab re-administration, and finally, three cycles of single-agent pemetrexed. Despite these treatments, the patient passed away 22 months after the initial diagnosis.

## Discussion

Outflow obstruction at the esophagogastric junction is frequently caused by invasion of esophageal, gastric, or esophagogastric junction cancers. However, when a patient presents with similar symptoms but shows no neoplastic changes in the esophagogastric mucosa on esophagogastroduodenoscopy, as seen in the current case, several differential diagnoses should be considered. These include achalasia and, in certain cases, even malignancies [3-7]. The patient presented with progressive dysphagia, weight loss, postprandial hiccups, and lower back pain, which raised suspicion of malignancy rather than idiopathic achalasia. CT revealed an irregularly shaped mass extending from the peri-esophagogastric junction to the retroperitoneum, along with pleural effusion, ureteral and hepatic lesions. These findings strongly suggested a malignant process.

Pseudoachalasia accounts for less than 5% of cases initially diagnosed as achalasia, with gastric adenocarcinoma being the most common cause [3-7]. Pseudoachalasia due to malignant pleural mesothelioma is exceedingly rare, with only a few reported cases. A systematic review of the literature reported that among 140 cases of pseudoachalasia, the most common underlying malignancies were gastric cancer (n = 27), esophageal cancer (n = 23), lung cancer (n = 19), and esophagogastric junction cancer (n = 16), while malignant mesothelioma was identified in only six cases [5,8-10].

Malignant pleural mesothelioma primarily originates in the pleura and is strongly linked to asbestos exposure [11-13]. In our case, imaging and endoscopic ultrasound findings suggested that pseudoachalasia resulted from direct invasion or extrinsic compression by the tumor extending to the peri-esophagogastric junction, rather than distant metastasis. This led to esophageal dysmotility due to mechanical obstruction and possible neuronal infiltration. Endoscopic biopsy alone often fails to detect malignancy, necessitating deep-tissue sampling via EUS-FNA for a definitive diagnosis. Despite chemotherapy, the median overall survival for advanced malignant mesothelioma remains limited to 12-18 months, with response rates varying among individuals [14].

Although pseudoachalasia is frequently linked to advanced and often incurable malignancies, it does not invariably indicate irreversibility or resistance to treatment. In some cases, especially those caused by paraneoplastic syndromes or reversible extrinsic compression, resolution may be achievable with appropriate treatment of the primary tumor [6,15]. Therefore, precise diagnosis of the underlying pathophysiology of pseudoachalasia is crucial to guide individualized management.

## Conclusions

This case highlights the importance of considering malignant mesothelioma as a potential, albeit rare, differential diagnosis in patients with progressive dysphagia and significant weight loss. Given the poor prognosis associated with advanced mesothelioma, early and accurate diagnosis is essential for guiding treatment decisions.
